# Optically Clear and Resilient Free-Form μ-Optics 3D-Printed via Ultrafast Laser Lithography

**DOI:** 10.3390/ma10010012

**Published:** 2017-01-02

**Authors:** Linas Jonušauskas, Darius Gailevičius, Lina Mikoliūnaitė, Danas Sakalauskas, Simas Šakirzanovas, Saulius Juodkazis, Mangirdas Malinauskas

**Affiliations:** 1 Department of Quantum Electronics, Faculty of Physics, Vilnius University, Saulėtekio Ave. 10, Vilnius LT-10223, Lithuania; darius.gailevicius@ff.vu.lt; 2 Department of Applied Chemistry, Vilnius University, Naugarduko Str. 24, Vilnius LT-03225, Lithuania; lina.mikoliunaite@chf.vu.lt (L.M.); danas.sakalauskas@chf.vu.lt (D.S.); simas.sakirzanovas@chf.vu.lt (S.S.); 3 Center for Micro-Photonics, Faculty of Engineering and Industrial Sciences, Swinburne University of Technology, Hawthorn 3122, Australia; 4 Melbourne Center for Nanofabrication, Australian National Fabrication Facility, Clayton 3168, Australia

**Keywords:** direct laser writing, ultrafast laser, 3D laser lithography, 3D printing, hybrid polymer, integrated micro-optics, optical damage, photonics, pyrolysis, ceramic 3D structures

## Abstract

We introduce optically clear and resilient free-form micro-optical components of pure (non-photosensitized) organic-inorganic SZ2080 material made by femtosecond 3D laser lithography (3DLL). This is advantageous for rapid printing of 3D micro-/nano-optics, including their integration directly onto optical fibers. A systematic study of the fabrication peculiarities and quality of resultant structures is performed. Comparison of microlens resiliency to continuous wave (CW) and femtosecond pulsed exposure is determined. Experimental results prove that pure SZ2080 is ∼20 fold more resistant to high irradiance as compared with standard lithographic material (SU8) and can sustain up to 1.91 GW/cm${}^{2}$ intensity. 3DLL is a promising manufacturing approach for high-intensity micro-optics for emerging fields in astro-photonics and atto-second pulse generation. Additionally, pyrolysis is employed to homogeneously shrink structures up to 40% by removing organic SZ2080 constituents. This opens a promising route towards downscaling photonic lattices and the creation of mechanically robust glass-ceramic microstructures.

## 1. Introduction

Hybrid organic-inorganic polymers have emerged as great materials for fabricating objects in both 2D and 3D configurations [1,2,3]. They are the material of choice for lithographic 3D femtosecond laser structuring due to several convenient features, which include optical transparency in the visible part of the spectrum [4] and the use of photoinitiators (PI) absorbing the UV radiation [5,6,7]. The latter makes them perfectly suitable for multiphoton polymerization [5,8] achieved by an ultrafast laser and employed in true free-form structuring by 3D laser lithography (3DLL) [9]. Additionally, their refractive index and mechanical properties can be tuned by changing the proportion between the organic and inorganic components [4]. This led to extensive research in this area and, to date, new hybrid materials containing Si [1], Zr [4] and Ge [10] were made for 3DLL.

The Zr containing hybrid photopolymer, mostly referred to as SZ2080, is especially interesting. It combines all of the best properties offered by these materials, such as low shrinkage, a hard gel form during fabrication, and transparency for visible light [4], and thus is widely used in creating structures to be employed in various applications in medicine [11], micro-optics [12] and photonics [13]. In the standard 3DLL case, photopolymerization of this material is initiated by nonlinear absorption in the PI molecule [14]. However, recent works showed that photopolymerization in SZ2080 can be achieved without PI using both tight focusing with high numerical aperture (NA) objective (NA > 1) and loose (NA < 1) focusing [15,16]. It is considered that this reaction is induced when nonlinear absorption takes place, which initiates the breaking of chemical bonds. Generated free electrons are subsequently accelerated by the intense electric field and provide bond cleavage via avalanche ionization [16,17]. This combination of multiphoton and avalanche ionization is responsible for subsequent crosslinking, which allows 3D microstructures to be formed out of pure material. Currently, it is known that SZ2080 in its pure form is biocompatible [11] and has a high optical damage threshold [18].

This paper aims at expanding knowledge of 3DLL with pure SZ2080. Special attention is given to its possible application in the field of micro-optics. Thus, experiments for determining the properties of pure SZ2080 relevant to this field are carried out. Results are compared to those obtained with photosensitized SZ2080. Functional micro-optical elements are manufactured and their resilience to continuous wave (CW) and femtosecond light exposure is tested. Furthermore, by applying pyrolysis, we remove the organic component of the hybrid material, leaving structures composed mainly of the glass-ceramic component. Finally, pyrolysis-induced shrinkage is employed in a controlled manner to create periodic lattices consisting of thin (∼170-nm-wide) sintered rods.

## 2. Results

First, the fidelity of 3D structuring of SZ2080 with and without PI was studied and microlenses were fabricated. Then, a comparative study of microlens performance with high irradiance was carried out down to the structural degradation level. Finally, sintering via pyrolysis aimed at retrieving glass-ceramic 3D structures with significant 40% size reduction was studied.

### 2.1. Comparison of Structuring Properties

**Figure 1:**
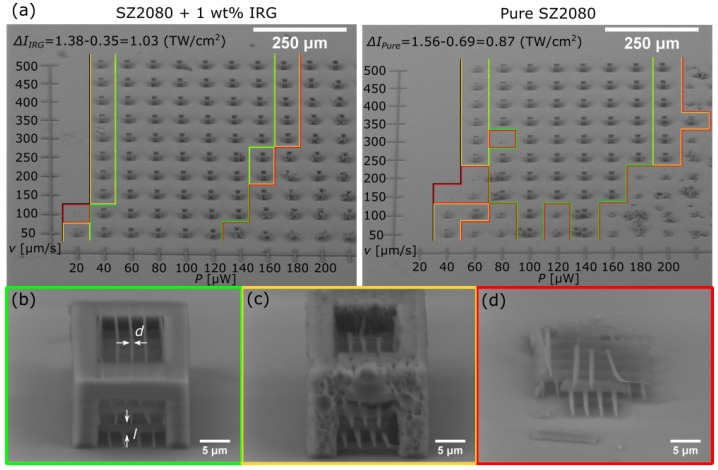
(**a**) SEM images of resolution arrays of photosensitized (**left**) and non-photosensitized (**right**) SZ2080. Structures with severe structural damage (**red**), with poor (**yellow**) and good (**green**) quality are outlined. The structure is considered good if internal single lines are observable and the shape of the cube is as designed. Average laser powers of the bottom and the top of the Δ*I* are recalculated to the peak intensity $I_{p}$ (shown at the top); (**b**) one of the good quality structures in the array is shown in a greater detail; *l* and *d* marks the longitudinal and transverse sizes of the lines; and (**c**) an example of a poor quality structure and (**d**) the failed one.

In 3DLL, a well chosen PI can improve fabrication throughput and structure qualities for the material used [6,7,19,20,21]. The set of parameters needed for structuring the material is generally referred to as the *fabrication window*. In essence, when all other experimental parameters are fixed, it can be considered to be an empirically determined intensity range Δ*I* between the irreversible polymerization threshold $I_{t}$ and $I_{d}$ at which the material is optically damaged: Δ*I* = $I_{d}-I_{t}$. We chose *I* (calculated using Equation (1)) as the main parameter to quantify the *fabrication window* instead of translation velocity/writing speed *v* because changes caused by the former are substantially less noticeable during experimentation than the deviations in structuring properties induced by even modest variations in the *I*. Conventional thinking would lead us to believe that forgoing photosensitization would lead to the absence of absorption, completely preventing photopolymerization or hindering it to the point of heavily inefficient crosslinking and narrow Δ*I*. In order to determine if that is the case, we designed an experiment in which an array of identical structures was fabricated by varying the *v* and *P*, as these are the two parameters that can be changed most practically during manufacturing. The structure chosen for this experiment was a cube with integrated single suspended lines. With this configuration, it is possible to determine several important factors. First, this array provides information about the size of Δ*I* by showing a structure survival rate dependent on the set of parameters used. The cube shows if it is possible to produce true 3D structures. Single lines give the possibility to measure fabricated feature sizes in transverse (*d*) and longitudinal (*l*) directions. For this reason, it is called a *resolution array*. The result outlined showing Δ*I* achieved with this experiment is provided in Figure 1.

**Figure 2:**
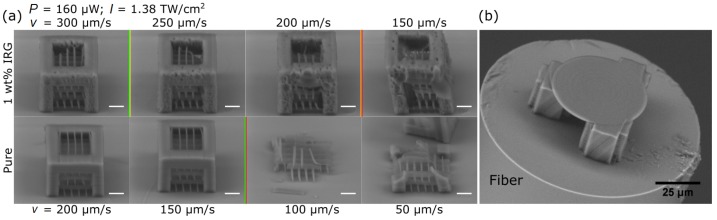
(**a**) reduction of structural quality in the case of photosensitized and pure SZ2080. Photoinitiator Irgacure 369 (IRG) containing cubes degrade slower and in a more progressive fashion. Conversely, the structures out of non-photosensitized material completely break up as soon as the fabrication parameters are not in the Δ*I*. All scales are 5 μm; and (**b**) monolithic micro-optical element on the tip of an optical fiber fabricated out of non-photosensitized SZ2080.

Data provided in Figure 1 shows several important features of pure SZ2080 in comparison to that containing PI. $I_{t}$, required to polymerize pure material, is higher by Δ$I_{t}=I_{\left(t\;pure\right)}-I_{\left(t\;IRG\right)}$ = 0.34 TW/cm${}^{2}$. The width of Δ$I_{IRG}$ is only 15.5% wider than Δ$I_{pure}$. By counting the sectors in which structures are of the best quality, we conclude that pure material provides only a 12.5% lesser survival rate compared to that of photosensitized polymer. In addition, the PI containing polymer provides structures that maintain their initial structural features even if parts of the object are greatly affected by the defects caused by overexposure, while in the case of objects formed from pure material, they completely collapse if non-optimal parameters are used (Figure 2a). This suggests that, without PI, the crosslinking process is not as efficient and provides a final polymer matrix that is considerably weaker. This result coincides well with other works showing that the degree of crosslinking during 3DLL is essential for the mechanical and optical properties of finished structures [22,23,24]. However, despite this, if fabrication parameters are within the Δ*I*, even advanced micro-optical elements, like suspended microlenses on the tip of an optical fiber, can be fabricated out of pure material (Figure 2b).

**Figure 3:**
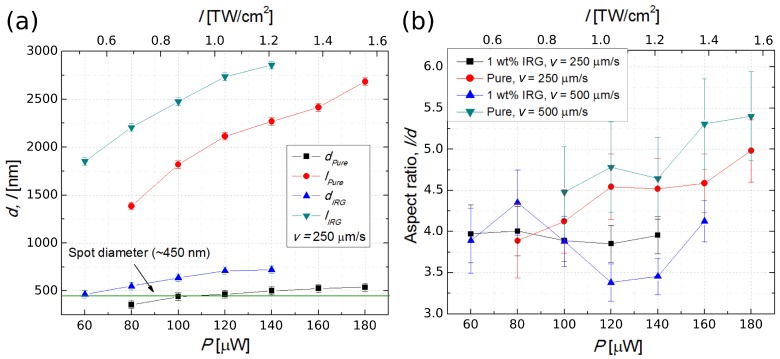
(**a**) feature width *d* and height *l* measured in the resolution array at writing speed of 250 μm/s; and (**b**) aspect ratio of lines produced for cases of 250 μm/s and 500 μm/s speeds.

Dimensions of the lines inside cubes were measured. The case of *v* = 250 μm/s was chosen as it is in the middle of the tested range in both photosensitized and non-photosensitized materials. It revealed that both transverse and longitudinal line dimensions are smaller in pure SZ2080 (Figure 3a). This correlates well with earlier findings [17]. It also reveals that features produced out of photosensitized material easily exceed the calculated spot size. On the other hand, lines produced out of pure SZ2080 are all about the same size as the focus spot. This can be explained by the fact that photochemical chain reactions, in the case of photosensitized material, can expand more easily out of the volume in which nonlinear absorption took place. In the case of pure resist, such a process is less prominent. In addition, the aspect ratios of the formed voxels are very similarly (Figure 3b), which shows that non-photosensitized material does not provide any benefit related to the control of the aspect ratio of a voxel. To better illustrate this, we provide data for the line aspect ratio for 500 μm/s writing speed in Figure 3b as well.

### 2.2. Surface Roughness

**Figure 4:**
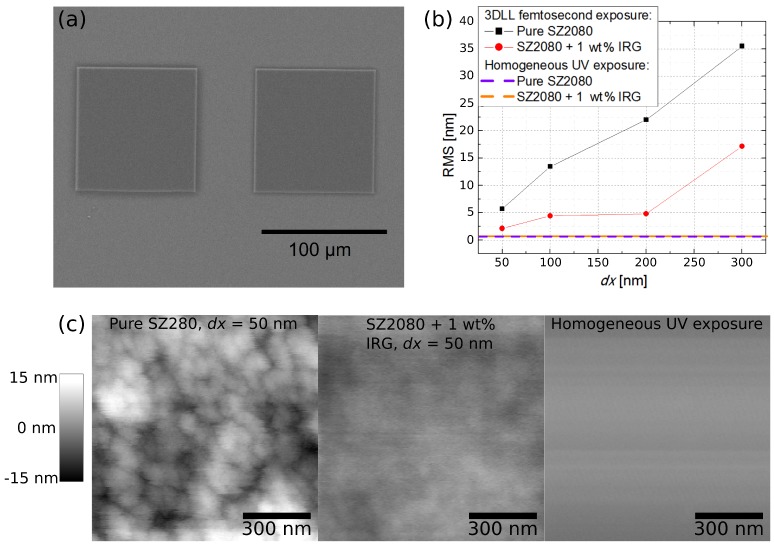
(**a**) SEM micrograph of the measured square polymerized structures; (**b**) RMS calculated for surfaces fabricated with different dx for SZ2080 containing PI and without it; and (**c**) AFM images of surfaces of pure and photosensitized polymer obtained with highest voxel overlap (dx = 50 nm) as well as one which was produced via homogeneous UV exposure.

Considering the application of SZ2080 in micro-optics, another important parameter is the surface quality of the final structures. There are several ways to quantify this property using data from precision measurement tools, such as an atomic force microscope (AFM) or very high magnification (>50 k) scanning electron microscope (SEM). The most common way to evaluate measured surface elevations is the standard root mean square (RMS), which was chosen for this study. It is common knowledge that if the surface roughness of a material is higher than *λ*/8, it is considered that the surface quality is insufficient for use in optics. On the other hand, if the roughness is smaller than *λ*/20, the material is considered suitable for optical applications. We are assuming micro-optical elements to be designed for use in the visible part of the spectrum, thus the lowest operational *λ* was chosen as 400 nm. AFM was employed to measure the surface profile of both pure and photosensitized SZ2080 samples. The geometry was of a flat square slab with side length of 100 μm (Figure 4a). Several different values of transverse voxel overlap (dx) were used to establish at which condition it was sufficient to achieve optical grade quality of the finished structure. It is important to note that our goal was to determine if optical grade surface quality can be achieved at all with parameters similar to those applied in microlens fabrication and, if so, whether it is easier to obtain it with photosensitized or pure polymers. For a control/comparison, we used a slab produced with one photon polymerization via homogeneous radiation of IV harmonic of an Nd:YAG laser (*λ* = 266 nm) similar to the one used in laser-induced damage threshold (LIDT) experiments [18,25]. After UV exposure, the samples were also submerged in the developer following the same protocol as laser-produced samples. This ensured that any difference in surface profile resulted from the polymerization method and not from the sample preparation.

Both polymers had a surface acceptable for optical applications. With the IRG containing material, surface roughness of RMS < 20 nm can be achieved with a smaller voxel overlap (dx = 300 nm, RMS = 17.1 nm), while in the case of pure polymer, the required dx is 100 nm (RMS = 13.5 nm) (Figure 4b). The femtosecond laser structured pure SZ2080 is inherently rougher when manufacturing parameters are taken from the middle of the Δ*I*. Even at dx = 50 nm, when the surface details of photosensitized SZ2080 become smooth, a non-photosensitized resist still exhibits clear nanofringes (Figure 4c). This could be explained by the fact that, with the non-photosensitized SZ2080, the polymerization mechanism is more chaotic, due to different and random process initiation pathways, compared to the photosensitized sample. It would also explain why such microstructures lose mechanical integrity much more quickly when the applied parameters are outside Δ*I*. It should be noted that a difference in roughness is induced by employing 3DLL, as the control samples of both IRG containing and pure SZ20820 polymerized by UV radiation showed the same flatness with RMS being less than 1 nm.

### 2.3. Resilience of Micro-Optics to High Irradiance

We next tested how functional micro-optical elements would perform under different light radiation conditions. It is known that pure SZ2080 should have a higher optical damage threshold when thin films characterized by standard LIDT [18,25]. However, it is still unclear how this translates into the operation of standard microlenses both qualitatively and quantitatively.

**Figure 5:**
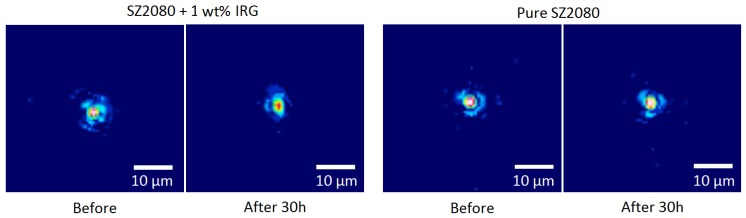
Images of the focal plane of a microlens before and after 30 h of exposure to 405 nm CW laser radiation. No significant change in the image at the focal point can be discerned.

First, an experiment was carried out with a CW *λ* = 405 nm laser operating at *P* ∼ 17 mW. The laser beam was focused to a *w* ∼ 250 μm radius spot onto 50 μm diameter microlenses, resulting in average intensity $I_{A}$ = *P*/*π*$w^{2}$∼ 8.66 W/cm${}^{2}$. The microlenses were produced following the procedure described earlier [26]. Intense laser radiation could induce changes both in the volume and on the surface of the micro-optical element. For this reason, focusing properties prior and after exposure to potentially damaging light irradiation were examined using a CCD camera to determine whether the microlenses were affected. Microlenses were left in 405 nm light for 30 h. The light source used to measure the focusing properties of microlenses in this experiment was an HeNe laser. As shown in Figure 5, with a CW UV laser operating at 405 nm wavelength and exposing the lenses for 30 h, no effects on the microlens focusing were observed.

**Figure 6:**
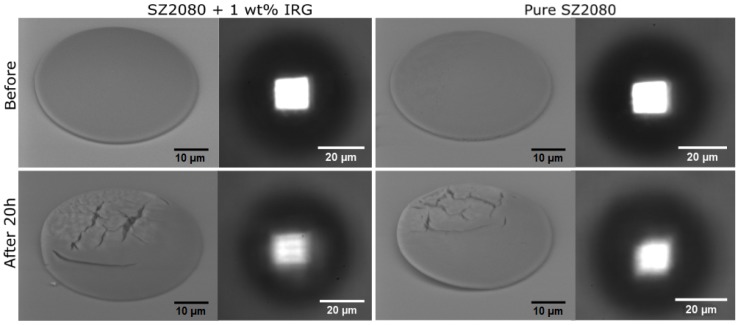
SEM images of microlenses before and after 20 h exposition to a loose focusing of 515 nm 300 fs laser radiation and an image of an LED made by the lens. Degradation of a lateral light distribution in the focal plane can be seen both in the structural quality of the lenses and the degraded projected image. The PI containing microlenses were more degraded.

Next, microlenses were left for 20 h exposed to a pulsed laser beam operating at 300 fs, 200 kHz and 515 nm wavelength. The microfabrication setup was applied for this experiment because it offered the possibility of both controlling the femtosecond laser beam irradiation parameters and simultaneously monitoring the focusing of the microlenses via the built-in microscope. A 40× NA = 0.95 objective was employed for imaging the microlenses as well as to provide focusing for 515 nm radiation. An LED was imaged through the lenses as an illumination source. The objective was retracted 85 μm from being directly focused on the micro-optical elements, thus allowing the laser beam to expand and to form a laser beam spot of ∼250 μm radius and $I_{p}$ = 0.85 GW/cm${}^{2}$. Furthermore, it was deliberately offset in a transverse direction by 55 μm from the center of the microlenses in order to see if damage to the microlenses would depend on the *I* of the laser beam. Such prolonged exposure to femtosecond laser pulses resulted in severely damaged microlenses, which showed changes in focusing properties and the overall integrity of the structure (Figure 6). This investigation of microlens focusing shows that a micro-optical element made out of pure SZ2080 suffered less damage.

**Figure 7:**
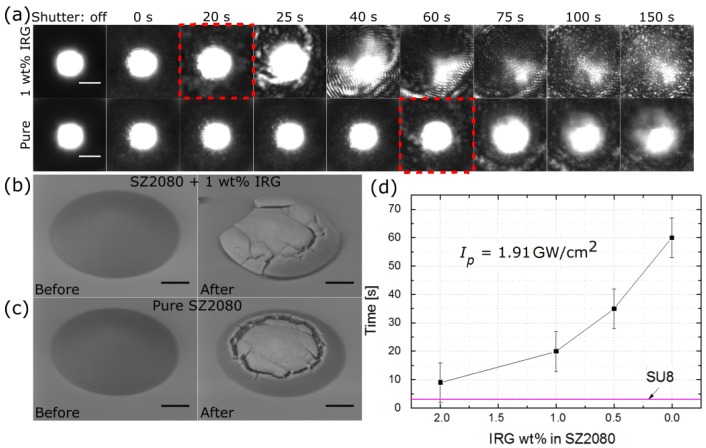
(**a**) real-time monitoring of a lateral intensity distribution of LED through microlenses produced using photosensitized and pure SZ2080 during irradiation with 515 nm 300 fs pulses at 200 kHz with $I_{p}$ = 1.91 GW/cm${}^{2}$ (spot radius of 100 μm). Faster deterioration of SZ2080 containing 1 wt % IRG as compared with pure SZ2080 is evident, as the image at the focus starts to degrade after 60 and 20 s (marked by **red** dashed squares), respectively; (**b**,**c**) SEM micrographs of the tested lenses before and after exposure. The photosensitized element is entirely destroyed, while the one produced out of pure SZ2080 exhibits relatively low damage; and (**d**) start of the microlens degradation for different concentrations of PI in SZ2080, as well as time needed to damage SU8. All scale bars are 10 μm.

In order to determine whether micro-optical elements of pure SZ2080 are indeed more resilient to intense laser radiation, the following time-dependent experiment was carried out. We used the 100× NA = 0.9 objective to in situ monitor changes in the focal plane of the microlenses. As the objective was retracted 50 μm from the microlenses, a ∼100 μm radius laser beam spot was formed, with $I_{p}$ = 1.91 GW/cm${}^{2}$. This exposure resulted in fast degradation of the image (Figure 7a). Two steps were discernible: when lens degradation becomes observable and when the LED image is completely obscured. In the case of lenses containing standard 1 IRG wt %, deterioration started after 20 s of irradiation and caused total destruction after 30 s. In the case of pure SZ2080 microlenses, the time period up to the beginning of deterioration was 60 s and 100 s to a fully obscured LED image. Hence, a microlens made out of pure SZ2080 can withstand about a three times larger exposure dose. Furthermore, the damage to the microlenses differed. The entire microlens was structurally damaged in the case of SZ2080 with 1 wt % IRG (Figure 7b) resembling a thermomechanical failure, while pure SZ2080 lenses were damaged only in the central region by homogeneous melting (Figure 7c). The experiments were repeated several times and showed that the result deviated by no more than 7 s from one experiment to another, which is in the range of tens-of-percent from the measured values. For better comparison, more tests were performed varying IRG concentrations (0.5 wt % and 2 wt %) and standard lithographic material SU8 (Figure 7d). Results proved that an increase in PI concentration leads to less time being needed before the laser light damages the microlens, namely 9 s with 2 wt % IRG. The non-hybrid SU8 started to deteriorate just ∼3 s after the shutter was opened with no noticeable deviations in time between the experiments. This shows that, even with relatively high (2 wt %) IRG concentrations, SZ2080 can withstand intense laser radiation at least three times longer than SU8. Removal of PI increases this superiority by more than one order (∼20 times), which agrees well with earlier findings in thin films [18]. Thus, in the case of intense continuous exposure, the hybrid material without PI is the best for microlens fabrication in terms of optical resiliency.

**Figure 8:**
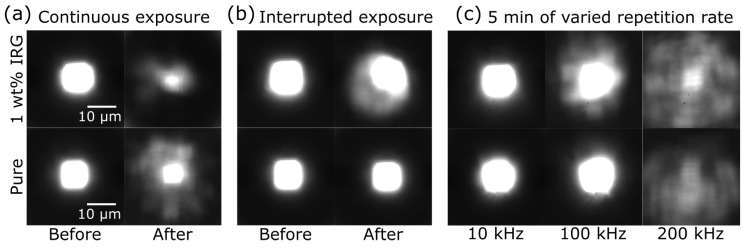
Focusing performance of microlenses after exposure to $I_{p}$ = 1.27 GW/cm${}^{2}$ 515 nm 300 fs radiation in continuous (**a**) and multi-burst mode: 10 s exposure followed by a 10 s pause (**b**); (**c**) lateral distribution at the focus of microlenses exposed to 5 min of continuous radiation at $I_{p}$ = 1.91 GW/cm${}^{2}$ (515 nm 300 fs) achieved with repetition rates of 10, 100 and 200 kHz. The 10 kHz case also serves as a before image, as there were no changes in focusing properties after this experiment.

The analysis presented shows degradation of micro-lenses during exposure. Whether it is caused by the accumulated dose or when critical temperature is reached was not established. Next, one set of lenses was continuously exposed for 15 min $I_{p}$ = 1.27 GW/cm${}^{2}$ radiation while the other set was irradiated for a combined 15 min exposure delivered in 10 s light bursts followed by 10 s pauses (30 min total). This resulted in the microlenses being severely damaged for both pure and photosensitized SZ2080 when exposed in a continuous manner (Figure 8a). However, the multi-burst exposure of non-photosensitized microlenses showed no noticeable distortions, while the photosensitized lenses were unmistakably degraded (Figure 8b). To further explore this effect, lenses were exposed to 5 min of continuous 1.91 GW/cm${}^{2}$ radiation with pulse repetition rates of 10, 100 and 200 kHz (Figure 8b). With 10 kHz illumination, both pure and photosensitized SZ2080 samples showed no noticeable changes in lateral light distribution at the focal plane, while both types of microlenses were completely destroyed by 200 kHz radiation. For this reason, repetition rates below or over these values were not tested. The response to 100 kHz varied between non-photosensitized and IRG containing SZ2080 microlenses. The effect on the pure SZ2080 microlens was inconspicuous, while the lens containing IRG displayed appreciable distortions. All these results clearly demonstrate that, in the case of pure SZ2080, the cause of damage is the heat accumulated in the volume of the lens. A better mitigation of the heat load could solve the thermal degradation of micro-optical elements.

### 2.4. Freeform Ceramic Structures out of Sintered SZ2080

Despite being more rigid and optically resilient in comparison to standard photopolymers [18], hybrid materials still have the limitations imposed by their organic component. On the other hand, the organic component is the reason why freeform laser nano-structuring is possible. Recent developments in the field of material processing showed that hybrid materials can be processed via pyrolysis by removing the organic component and leaving only the ceramic structure. This was successfully demonstrated using stereolithography at a millimeter-scale [27,28] or at a smaller scale using commercially available photoresists [29,30]. Here, we show a similar result in the micro- and nano-scales for SZ2080.

**Figure 9:**
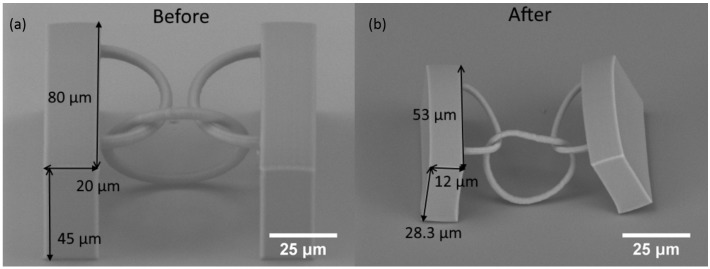
SEM images of the structure consisting of supporting walls and free hanging ring before (a) and after (b) pyrolysis. Magnification is the same. Fabricated objects appear brighter after pyrolysis, which indicates a change in the electrical conductivity of the material.

The initial sample structures chosen for the experiment were thick supporting walls with free hanging chain between them (Figure 9a). The sintered structure shrunk to about 65% in respect to all initial dimensions (Figure 9b). This indicates homogeneous reduction in sizes and volume during pyrolysis. Thus, in further experiments, we considered that the measurement of change in one dimension is sufficient to determine the overall change in the volume. In addition, the free hanging ring shrunk and deformed unevenly during pyrolysis. Peeling of supporting structures from the substrate was observed.

**Figure 10:**
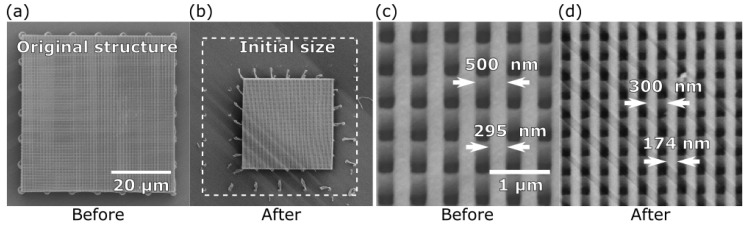
(**a**) SEM micrograph of a photonic crystal prior to (**a**) and after (**b**) pyrolysis; (**c**) the period and line width initially were 500 nm and 295 nm, respectively, which were shrunk to 300 nm and 174 nm after pyrolysis (**d**); a change of ∼40%. The period/width ratio stayed ∼1.7, indicating a homogeneous reduction in size.

Next, pyrolysis was employed to tailor photonic crystals with ultra-thin lines. Photonic crystals designed to operate in the visible part of the spectrum require their resolution to be sub-wavelength [31]. 3DLL allows one to achieve the needed feature sizes [32,33], yet is relatively problematic due to the necessity of using ultra-short (<100 fs) laser pulses, tight focusing with oil-immersion lenses, and complicated post-processing techniques, such as critical point drying [34]. Here, we produced woodpile photonic crystals with line widths that can be achieved without any additional post-processing and then reduced in size by the application of pyrolysis. Furthermore, 295 nm wide lines (Figure 10a) were changed to 174 nm (Figure 10a), which is a reduction of ∼40%. The processed structure retained its overall shape. This is demonstrated by the ratio between the photonic crystal period and line width retaining the same ∼1.7 value before and after pyrolysis. Therefore, this technique does not require complicated compensation algorithms to be used during direct laser writing (DLW).

**Figure 11:**
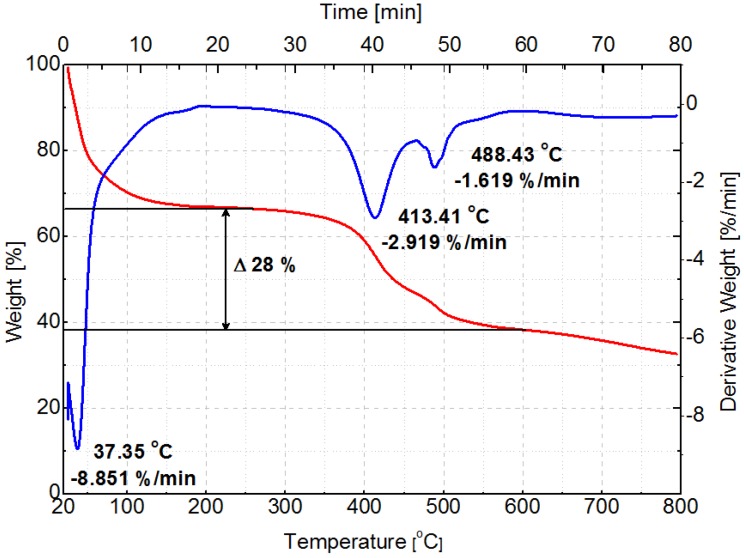
TGA data showing weight loss vs. temperature. The weight loss was 28%; observed shrinkage was ∼40%. The organic component was decomposed and removed by heating.

In order to gain further insights into what happens during pyrolysis, a thermal gravimetric analysis (TGA) of a drop of unprocessed SZ2080 was performed (Figure 11). First, the sudden drop in the weight at 100 ${}^{\circ }$C can be attributed to the evaporation of solvent that otherwise would be removed during pre-bake. The weight then remained relatively stable until the temperature reached 350 ${}^{\circ }$C, when it started again to decrease rapidly. The organic component of the hybrid is decomposed at this stage. During this period, two distinct changes in the weight decrease are discerned, hinting at two phases in which the organic component of the material is removed from the hybrid. The overall change in weight was about 28%. This exceeded the volume change of ∼35%–40% and indicates that the lost part of the material is relatively low-density organic compounds. In addition, it is assumed that the remaining material is densified, resulting in a shrunken ceramic structure.

## 3. Discussion

**Figure 12:**
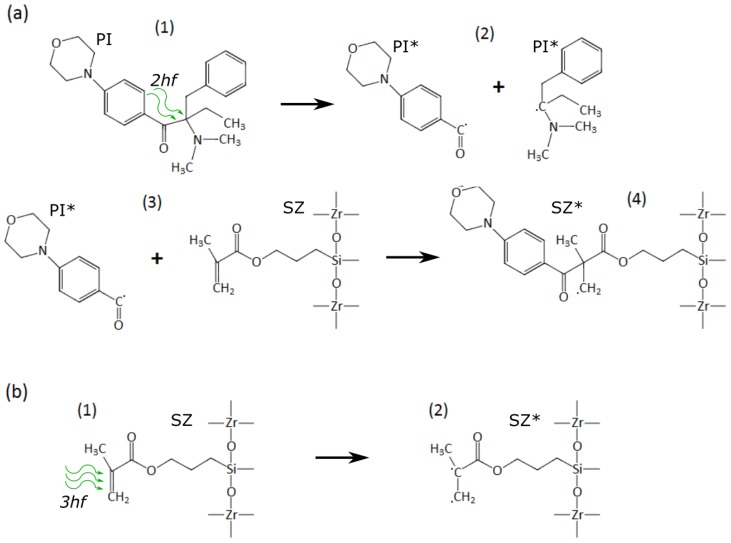
(**a**) polymerization reactions initiated by nonlinear absorption of PI molecules and subsequent chemical pathways resulting in a cross linked SZ2080; (**b**) SZ2080 cross linking without PI. *hf* is the photon energy.

Here, we discuss the 3DLL of pure materials in detail. First, femtosecond laser photopolymerization of a SZ2080 with IRG PI is recalled. Light breaks the weak single bonds of the PI molecule, which then creates two radicals PI* (Figure 12a (1,2)) [17] . These radicals then react with pre-polymer molecules via double bonds creating a radicalized monomer SZ* (Figure 12a (3,4)). This initiates a chain reaction and growth of an intertwined polymer matrix, which does not dissolve in the organic solvent. In the case of a SZ2080 without PI, such a reaction is induced when nonlinear absorption occurs. The double bond is broken in the pre-polymer (Figure 12b (1,2)), which otherwise would be broken by reactions with PI molecules. Large excitations can build up in the intermediary states because the laser-induced multiphoton excitation rate of pre-polymer species is high in comparison to the thermalization rate, which can be as long as μs [35]. Photochemical (photolytic) processes are dominant compared to photothermal phenomena due to the absence of detectable thermal effects, such as sample vaporization, boiling or thermal expansion, which is ensured by the closure of the reaction volume by the surrounding material. High irradiance exposure is sufficient to directly break chemical bonds of pre-polymerized SZ2080 organic constituents, which initialize formation of an insoluble and rigid organic-inorganic composite.

The difference of Δ*I* for both pure and PI containing material being only 15.5% is an excellent result, showing that the application of femtosecond pulses and NA = 1.4 focusing is sufficient to make experimentation with both materials relatively easy. However, in order to tie this parameter to a more diverse set of materials and setups, we need to normalize it to the $I_{t}$, thus acquiring a dynamic fabrication range: DR = Δ*I*/$I_{t}$ [36]. This allows us to describe manufacturing conditions in terms of position and width of Δ*I* in relation to applied *I* and $I_{th}$, which is heavily influenced by all fabrication parameters. In the case of our study, $DR_{IRG}$ = 2.94 is more than two times higher than $DR_{Pure}$ = 1.26. This result is a consequence of pure material being less photosensitive (higher photon densities are required for avalanche induced direct bond breaking than multi-photon absorption) and having twice as high $I_{t}$. Lower DR indicates that, while processing such polymers, special care and skills should be taken when finding the structuring parameters. It is important to note that the two-fold discrepancy in DR is substantially higher than the 15.5% difference in Δ*I*. Thus, DR is more universal in demonstrating sensitivity to applied fabrication parameters than case-sensitive Δ*I*. Universal parameters of this kind are advantageous when comparing results achieved in other fabrication setups with different materials and light sources, such as CW lasers [37,38], ps pulses [39] or high repetition rate Ti:Sapphiresystems [36]. On the other hand, this particular parameter still has limitations considering its usage with diverse pulse overlaps caused by variation in *v* or pulse repetition rate. Further research aimed at better understanding the processes involved in nano-structuring and its numerical evaluation could be one of the directions for further work in the field.

With ever-increasing demand for advanced elements in optical systems, freeform 3D microstructuring techniques become more sophisticated. While advanced glass processing methods show some possibilities for producing relatively small (∼nm–μm) structures [40], they still lack insurface quality and the capability of performing true 3D fabrication. To date, only (DLW) based 3DLL was shown to be capable of true 3D structuring at the microscale. This additive manufacturing technique [41] combines the complete freedom of the architecture of produced objects [42], the possibility of integration on various substrates [43,44,45] and a large range of materials that can be processed in such a fashion [46]. Here, we have shown that structures produced out of zirconium containing photopolymer SZ2080 can withstand a relatively low $I_{A}$ = 8.66 W/cm${}^{2}$ short wavelength (405 nm) exposure for prolonged time periods. Additionally, it was demonstrated that micro-optical elements made out of SZ2080 have good resilience to high repetition rate femtosecond laser radiation. This matches well with earlier findings of higher optical damage threshold for SZ2080 [18]. Finally, we have shown that the surface quality of structures produced from non-photosensitized and photosensitized SZ2080 has low surface roughness (RMS < *λ*/20 at *λ* < 400 nm) and is suitable for applications in micro-optics and opto-fluidics [47,48].

The development of micro-optical elements made from a high-purity glassy hybrid material via a simple low temperature chemical synthesis without use of the PI, which are usually required for light absorption, can help new developments in several fields. For high-intensity laser applications, the use of micro-optical elements has several distinct advantages due to optical damage scaling rules [49]: (1) the damage threshold decreases with increasing spot area $I_{dam}\propto 1/\sqrt{A_{spot}}$; (2) micro-optical elements have low surface roughness, $\delta_{surf}$, which increases the damage threshold $I_{dam}\propto 1/\delta_{surf}^{m}$ with exponent *m* between 1 and 1.5. In the field of light filamentation of ultra-short laser pulses in air and gases, the exploration of multiple beamlet generation apertures [50], ring Airy beams [51], and the realization of multifilaments– super-filamentation [52] is an active research area. The peak intensities on the beam-forming optics can reach pre-breakdown *I* of several TW/cm${}^{2}$, which demands low absorption materials (no PI in polymerised micro-optics) according to the scaling $I_{dam}=\frac{0.26\;\mathrm{MW}/{\mathrm{cm}}^{2}}{{\left(\alpha /{\mathrm{cm}}^{-1}\right)}^{0.74}}$, where the absorption coefficient α=−1/Lln(T) is defined by transmission *T* and propagation length *L* [49]. While microlenses have an inherently low *L*, the result of removing PI also greatly minimizes *T*. This is the reason why small optical elements using refractive and diffractive beam-forming concepts made from high purity materials are the most promising candidates in the high-power laser field.

The fact that material can be structured without PI with a Δ*I* comparable to that of photosensitized material is a promising discovery for other science fields as well. Earlier studies dedicated to pure material structuring by femtosecond laser pulses [16,53] have proved complicated and slow. If such pure material can be processed at a relatively high speed (∼mm/s), it would become suitable for biomedical applications, where structure dimensions are in the range of mm–cm [54]. Pure material would guarantee superb biocompatibility, which is a key requirement for tissue engineering, especially taking into account biodegradable implants.

For other diverse applications, SZ2080 can be doped with organic die molecules [55] or noble metal nanoparticles [56], which can be used for polymerization control or increased functionalities. In the recently developed field of astro-photonics [57], where absorption in laser written waveguide lanterns is strong [58], the polymerised waveguides can tackle high loss problems. The photonic wire bonding also improves light confinement, flexibly controls 3D conformation of fiber bundles for phase matched light delivery from the optical image plane to a spectrometer slit, and well defines single-mode operation of the fiber by precise control of the polymerised wire cross section. Unexpectedly, from the perspective of high light intensity applications, large volume optical astro-instrumentation is highly sensitive to detector background counts due to radioactive trace elements in the glass-optics. Potentially, better control of glass forming ingredients can be obtained in the case of photopolymer selection for optical elements prepared via a sol-gel route as SZ2080.

Composition control of the sol-gel resists via different portions of organic and inorganic components provides a tool to tune the refractive index [4] and to create complex micro-optical elements for high-quality optical imaging, as compared with only shape control of composite lenses [44,59,60,61]. Since the sol-gel route is open to mixing different oxide precursors and concentrations, this opens up the capability of creating the different refractive index PI-free optical elements required for aberration control in multi-component lens optical systems [44,59,60,61]. With pyrolysis, an even larger range of refractive index tunability is accessible. Current endoscopy and optical imaging applications where micro-optical elements are in contact with live tissue would benefit from the absence of PI due to strong optical absorption and bio-toxicity.

The possibility of using pyrolysis is an additional feature of SZ2080 due to its hybrid organic-inorganic nature that allows it to achieve true 3D glass ceramic structures. This is expected to open up new applications. Shrinkage of the polymer can be used to achieve ultra fine features. The demonstrated homogeneous reduction in size by 40% while keeping a well-defined 3D structure is an improvement compared to the 30% presented in other work [30]. Further studies aimed at better understanding the underlying physical and chemical phenomena during pyrolysis, as well as enhanced control of SZ2080 sintering, will be the basis for our future work.

## 4. Materials and Methods

The SZ2080 photoresist was acquired from FORTH (Heraklion, Greece) and, as its name implies, contained 20 wt % of inorganic and 80 wt % of organic components. For cross-check experiments, SZ2080 was photosensitized by mixing it with a commercial PI Irgacure 369 (IRG). Samples were prepared by drop casting one droplet of the material on a glass substrate and then pre-backing the sample at 75 ${}^{\circ }$C for 45 min. After fabrication, samples were developed in isobutyl methyl ketone for 45 min and subsequently rinsed in isopropanol for 15 min. The SU8 was processed using a procedure consisting of two pre-bake stages, 30 min at 60 ${}^{\circ }$C and 60 min at 90 ${}^{\circ }$C, and then post-baked at similar temperatures but with half the duration (15 and 30 min, respectively) and developed in propylene glycol monomethyl ether acetate (PGMEA) for 60 min.

**Figure 13:**
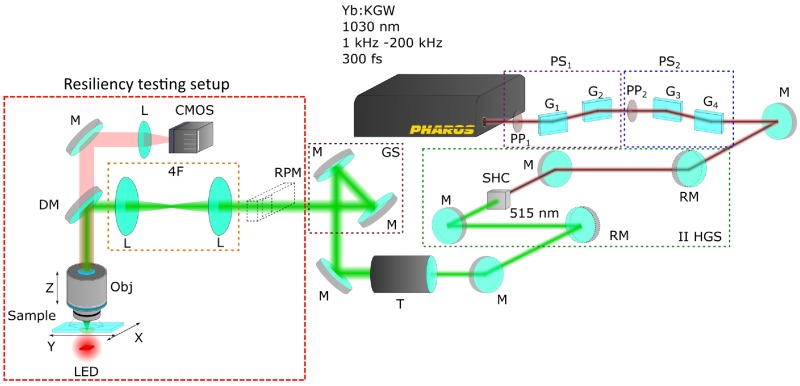
Schematics of setup used for fabrication and microlens degradation experiments: PS—power control stage, PP—phase plate, G—glass plate, M—mirror, RM—removable mirror, SHC—second harmonic crystal, T—telescope, GS—galvanoscanner, RPM—removable power meter, L—lens, 4F—lens system in 4-F configuration, DM—dichroic mirror, CMOS—CMOS camera used to monitor fabrication process, Obj—objective lens, LED—LED used for sample illumination.

Schematics of the 3DLL setup used are shown in Figure 13. The femtosecond laser was Pharos (Light Conversion Ltd., Vilnius, Lithuania) operating at 1030 nm fundamental wavelength, 300 fs pulse duration and 200 kHz repetition rate. Power is controlled by two power control units consisting of a *λ*/2 waveplate and Brewster angle polarizer. Such two-stage power attenuation allows for minimizing power fluctuations and provides precise power control during fabrication. A second harmonic at 515 nm wavelength was used for 3D free-form polymerization. The laser beam is expanded by a 2× magnification telescope in order to fill all of the objective aperture. During experiments in which precise feature size control was essential (for instance a photonic crystal), polarization of incident light was kept at a constant 45${}^{\circ }$ degree angle with both horizontal translation axes, this way avoiding any polarization induced anisotropy of fabricated line widths [62]. Structure fabrication is performed with a combination of Aerotech linear stages (ALS130-110-*X*,*Y* for positioning in the XY plane, ALS130-60-*Z* for *Z*-axis) and a galvanoscanner, operating in sync in an infinite field of view (IFOV) regime, which allows high fabrication speeds (∼mm/s) and superb structure quality. The sample is illuminated by a red LED which enables the fabrication process to be monitored in real time using the CMOS camera.

The same setup was used to monitor the degradation of micro-optical elements in real time. In this case, the sample was microlenses on a glass slide illuminated by the LED from the bottom. The objective was retraced at some defined distance from them, resulting in a relatively large laser spot on the lenses. Exact values are listed in the text where applicable. This allowed for monitoring lateral intensity distribution projected by the microlens and for illuminating them with femtosecond laser light simultaneously.

The average laser power *P* was measured before the polymerization and lens degradation experiments and subsequently recalculated at the peak intensity $I_{p}$ at the center of focal point [15]:

(1) $$I_{p}=\frac{2PT}{fw^{2}\pi \tau },$$

where *f* is the pulse repetition rate, *τ* is the pulse duration, and ω=0.61λ/NA is the waist (radius) of the beam. T≃0.41 is the system transparency without glass substrate and pre-polymer for a 63× NA = 1.4 objective. Fabrication parameters used for microlenses are $I_{IRG2\%}$ = 0.48 TW/cm${}^{2}$, $I_{IRG1\%}$ = 0.48 TW/cm${}^{2}$, $I_{IRG0.5\%}$ = 0.61 TW/cm${}^{2}$ and $I_{pure}$ = 0.61 TW/cm${}^{2}$ using *v* = 50 μm/s. For AFM analysis, structures were produced at $I_{IRG}$ = 0.61 TW/cm${}^{2}$ and $I_{pure}$ = 0.86 TW/cm${}^{2}$ and *v* = 250 μm/s. The latter parameters were used for fabrication of the sintered ring structure. The photonic crystal for pyrolysis experiments was acquired applying *v* = 100 μm/s and *I* = 0.84 TW/cm${}^{2}$. The Nd:YAG laser used for IV harmonic (λ=266 nm) generation operated at 5 ns pulse duration, 24 mJ pulse energy, 2 Hz repetition rate and was directed onto a 1 cm diameter spot. Both pure SZ2080 and with 1 wt % were exposed to such radiation for 30 min. Pyrolysis was performed in Ar atmosphere at 600 ${}^{\circ }$C temperature for 5 h.

Surface roughness was characterized using SEM TM-1000 (Hitachi, Tokyo, Japan) and AFM Catalyst (Bruker, Billerica, MA, USA) with an Au coated SiN-needle with a *k* = 0.06 N/m stiffness at *F* = 18 kHz and with a tip diameter of 20 nm. For TGA analysis, Pyris 1 TGA (Perkin Elmer, Waltham, MA, USA) equipment was used; the sample was heated in a nitrogen atmosphere from 30 ${}^{\circ }$C to 800 ${}^{\circ }$C with a heating rate of 10 ${}^{\circ }$C/min.

## 5. Conclusions

A detailed study of laser structuring of non-photosensitized SZ2080 via 3DLL was carried out. The photo-polymer has only a 12.5% lower structure survival rate compared to SZ2080 photosensitized with 1 wt % IRG and comparable Δ*I* ∼ 1 TW/cm${}^{2}$. Surface roughness of structures produced out of both compositions was in the range of RMS < 20 nm, which is sufficient for fabricating micro-optical components. The structuring fidelity of both materials is comparable if parameters from within the Δ*I* are used. A micro-optical element made out of pure SZ2080 was integrated on the tip of an optical fiber.

Furthermore, investigation of micro-optical element degradation at various laser irradiations was performed. It was shown that SZ2080 in both photosensitized and pure forms was not damaged by tens of hours of exposure to CW 405 nm laser providing $I_{A}$ = 8.66 W/cm${}^{2}$. On the other hand, microlenses produced out of pure material were shown to survive ∼three-fold longer in comparison to those containing 1 wt % IRG, and ∼20 times longer than those produced out of SU8 when irradiated by 515 nm 300 fs 200 kHz laser at $I_{p}$ = 1.91 GW/cm${}^{2}$. If $I_{p}$ is dropped to 1.27 GW/cm${}^{2}$ and delivered in an interrupted manner (10 s of exposure followed by 10 s pause), non-photosensitized microlenses showed no signs of degradation even after combined exposure of 15 min, while those containing IRG were damaged substantially. A similar result was achieved with a decrease in the pulse repetition rate, while keeping the $I_{p}$ = 1.91 GW/cm${}^{2}-10$ kHz caused no distortions in both pure and photosensitized structures, 100 kHz induced substantial damage only in those containing the IRG, and 200 kHz destroyed both types of lenses. This indicates that, in the case of microlenses, the main deterioration inducing factor is thermo-accumulation and subsequent melting.

Additionally, pyrolysis of hybrid material was performed removing organic constitutes, leaving only densified glass-ceramic structure. Shrinkage during this process was homogeneous and allowed for size reduction by 35%–40%, which is a record high number [30]. Further studies will be focused on the optical and mechanical properties of glass-ceramics and its applications for high irradiance optics and material processing with high $I_{p}$ of high-repetition rate conditions [63,64]. Potential applications are in the fields of the filamentation of ultra-short laser pulses, the fabrication of fiber-optical elements for sensor applications in the chemically harsh, high temperature, and radioactive environments encountered in nuclear power stations and in optically driven inertial confinement fusion facilities.
